# Preface to ‘Developing resilient energy systems’

**DOI:** 10.1098/rsta.2021.0131

**Published:** 2022-04-18

**Authors:** C. Richard A. Catlow, Legena Henry, Catherine Ngila, Michael Taylor

**Affiliations:** ^1^ University of Cardiff, UK; ^2^ University College London, UK; ^3^ The University of the West Indies, Cave Hill, Barbados; ^4^ African Academy of Sciences, Nairobi, Kenya; ^5^ The University of the West Indies, Mona, Jamaica

The need for resilience and sustainability in the systems on which Society depends is increasingly recognized as a global priority. It is also clear that to achieve resilience, input from science, technology and innovation will be essential. For this reason, the Royal Society in partnership with the African Academy of Sciences chose *Science for a Resilient Future* as the theme for the third Commonwealth Science Conference, which was held virtually in February 2021 and brought together 350 scientists (of whom 270 were early career) from 32 Commonwealth nations to discuss three key areas within the overall theme of science for resilience: *Developing resilient energy systems*; *Nurturing resilient ecosystems*; and *Building resilient societal systems.* The present volume, which comprises papers from participants at the conference, focuses primarily on the first theme, although with some discussion of topics relevant to the third. The second theme will be addressed in a separate volume of *Philosophical Transactions B.*

The development of resilient energy systems is essential for the control of climate change and in many parts of the world a necessary adaptation strategy. It is also critical for the development of sustainable and circular modes of production and consumption. As well as wide ranging scientific and technological challenges, including those relating to behavioural and social aspects, there is a complex interplay between science and policy; and both scientific and policy aspects are illustrated by several of the papers in this volume. The issues raised are, of course, global as again was clear from the conference and from the studies reported here. Indeed the Commonwealth as a network of both developed and developing nations provides an effective forum for the discussion of resilience in the global context. We hope that the work discussed in this volume will help to stimulate and develop global interactions and collaborations in this crucially important field.

We would like to express our thanks to the Steering Committee of the Commonwealth Science Conference, to all the speakers and participants in the conference and to the staff of the Royal Society and African Academy of Sciences for their work in organizing the conference on which this volume is based. The conference was funded by the UK Government Global Challenges Research Fund (GCRF).

